# Supplementary material for: Aldehyde-alcohol dehydrogenase forms a high-order spirosome architecture critical for its activity
Source: Nat Commun. 2019 Oct 4;10:4527. doi: 10.1038/s41467-019-12427-8 (PMC6778083; doi:10.1038/s41467-019-12427-8)
Supplement: Supplementary file 1 — Supplementary Information [file 41467_2019_12427_MOESM1_ESM.pdf]

Supplementary Information

Aldehyde-alcohol dehydrogenase forms a high-order spirosome architecture critical for its activity

Kim et al.

a

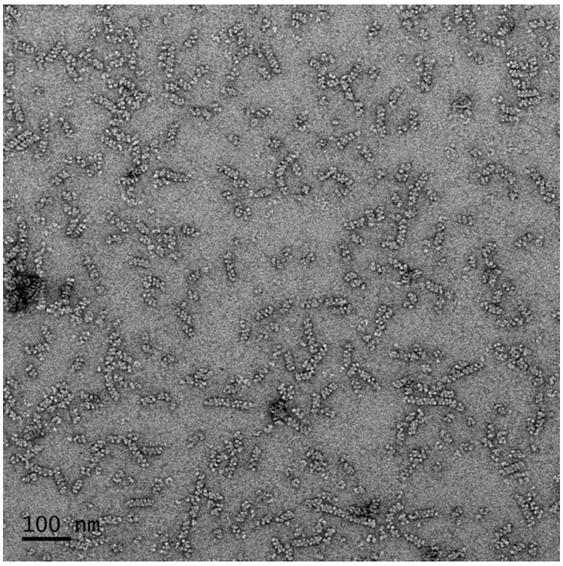

b

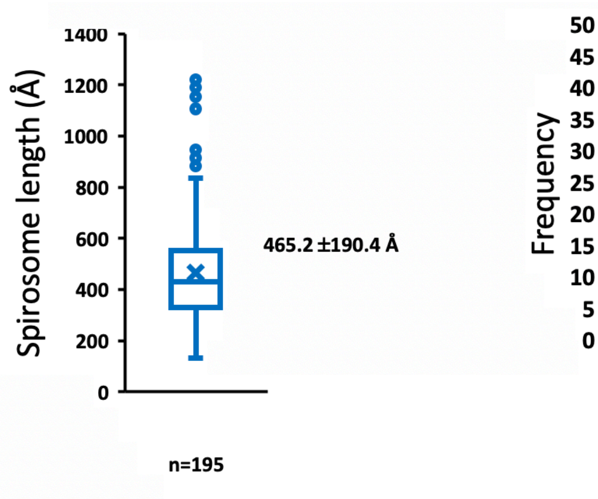

c

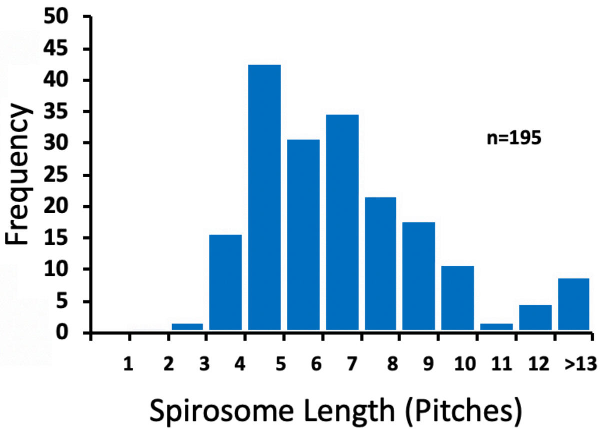

Supplementary Fig. 1. The length distribution of spirosomes. a. A representative negative-stained micrograph of AdhE. The box plot shows the length distribution of spirosomes. The box includes the inter-quartile range from Q1 to Q3. The x in the box indicates the median. **b.** Graph shows the average length (465.2 Å) of the spirosomes and the standard deviation (190.4 Å) (n=195). **c.** Bar graph of the distribution of spirosome in length in pitches (1 pitch corresponds to 70Å in length).

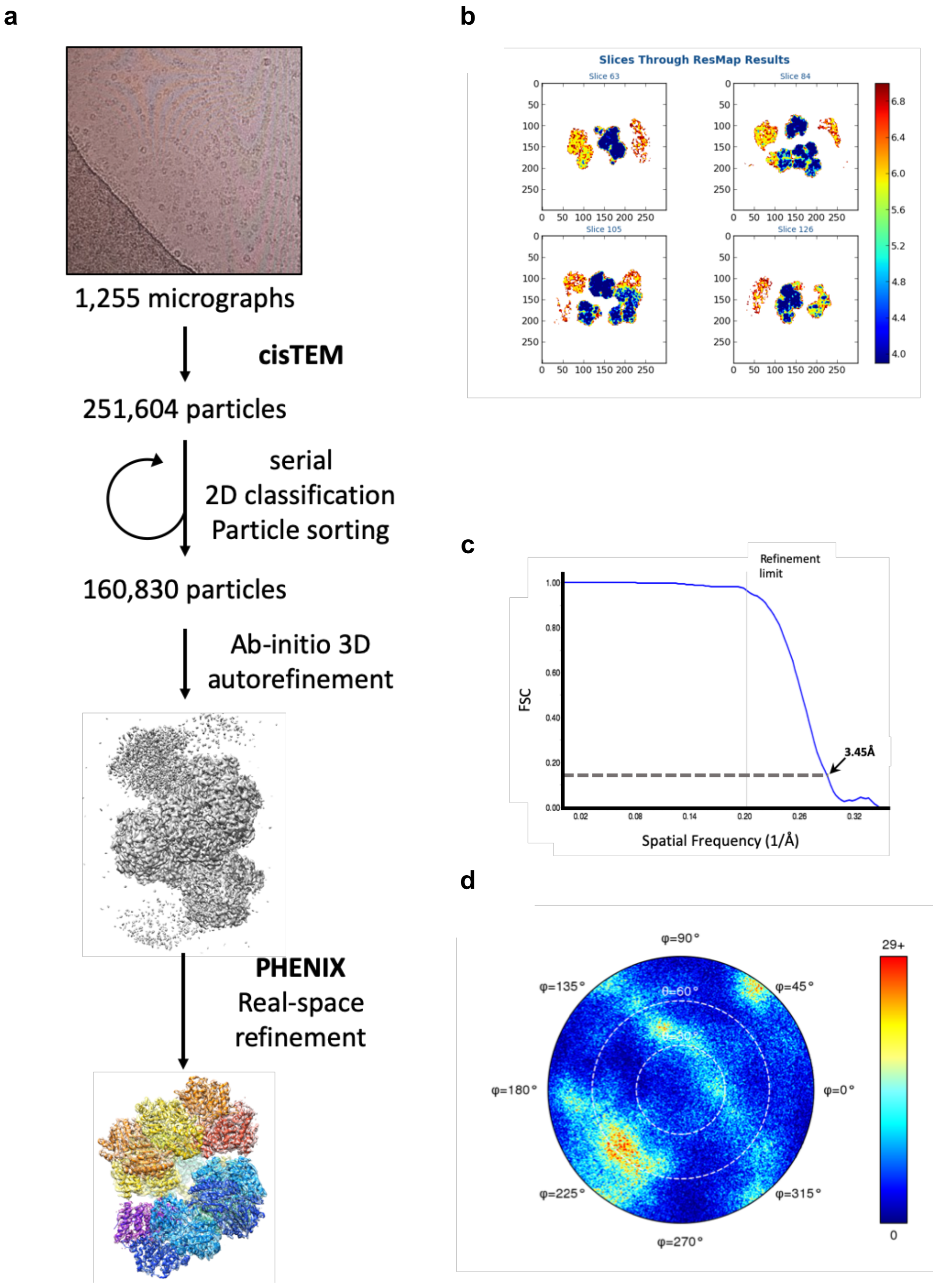

Supplementary Fig. 2. Cryo-EM structure determination. a. A scheme for cryo-EM image processing. **b.** Local resolution heat map of AdhE. Local resolution was estimated using ResMap[1]. The blue colour indicates higher resolution and the red colour lower resolution. **c.** Resolution was determined by an FSC coefficient criterion of 0.143. **d**. Euler angle distribution of the particles used for the reconstruction of the cryo-EM structure.

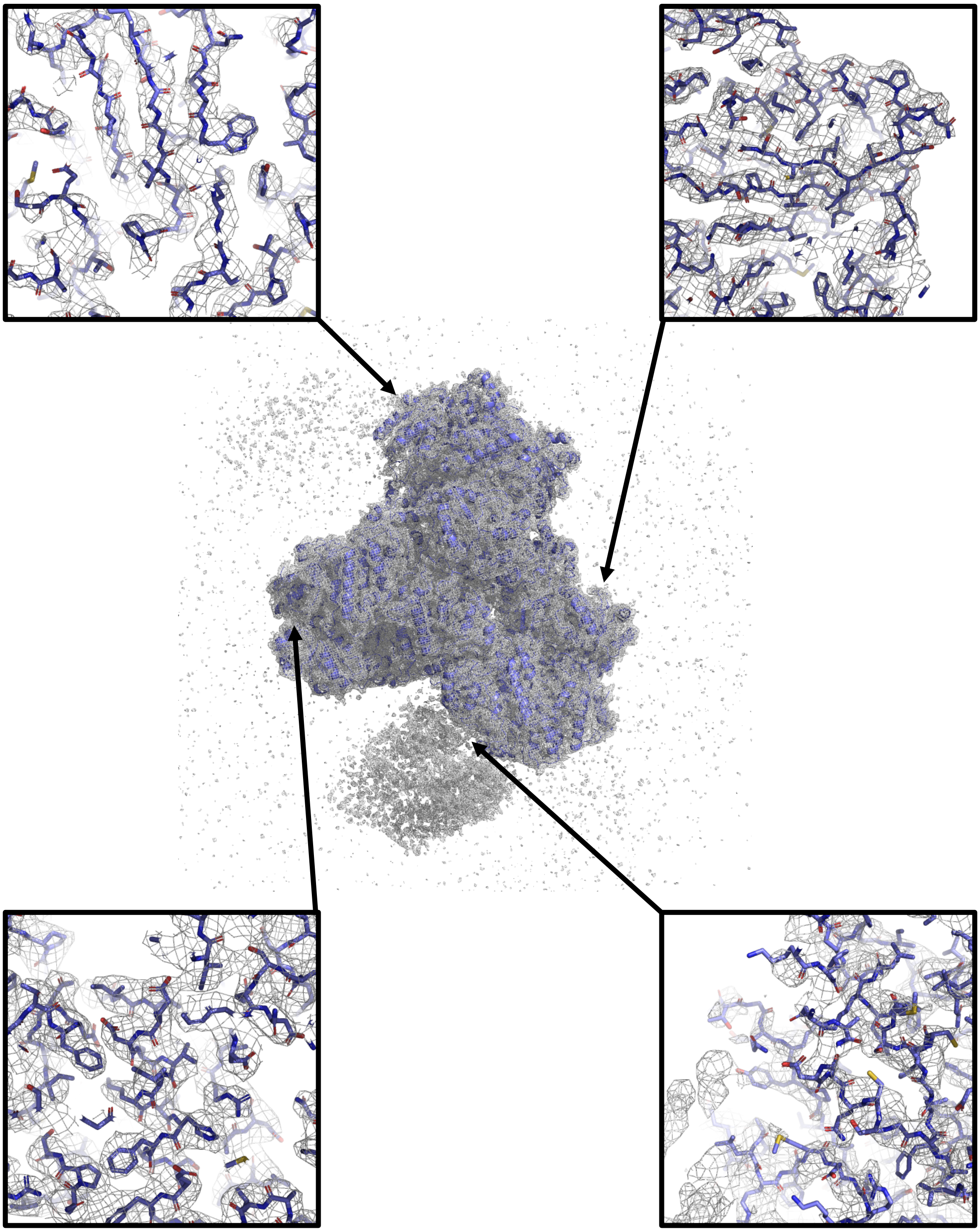

Supplementary Fig. 3. Cryo-EM maps of AdhE with atomic models. A Cryo-EM map of AdhE with a refined model.

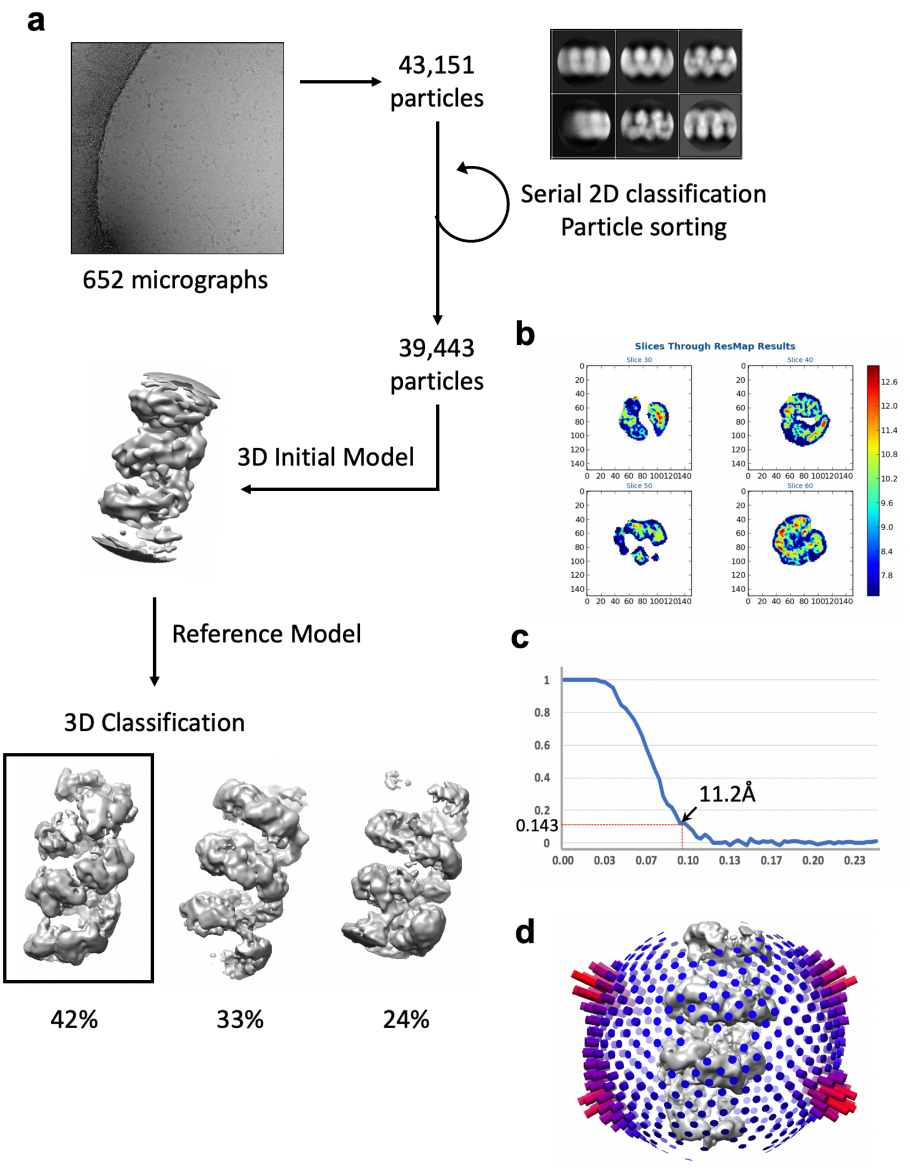

Supplementary Fig. 4. Helical reconstruction of AdhE. a. 3D classification of AdhE. **b.** Resolution was determined by an FSC coefficient criterion of 0.143. **c.** Resolution was determined by an FSC coefficient criterion of 0.143. **d**. Euler angle distribution of the particles used for the reconstruction.

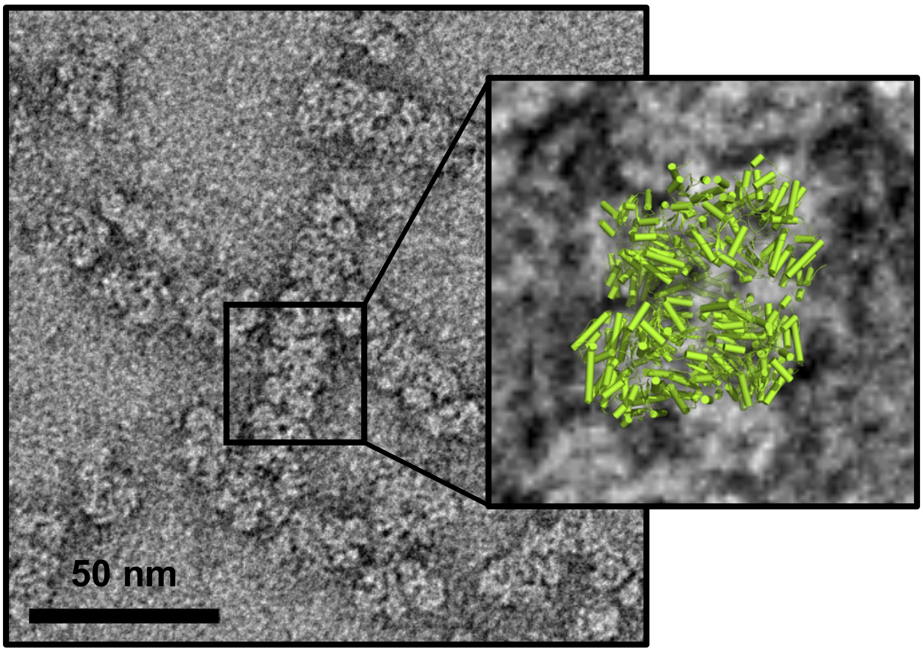

Supplementary Fig. 5. Overlaying the cryo-EM structure on a negative stain EM micrograph. The cryo-EM structure of AdhE molecules (represented by green) was manually superimposed on a negative stain EM micrograph.

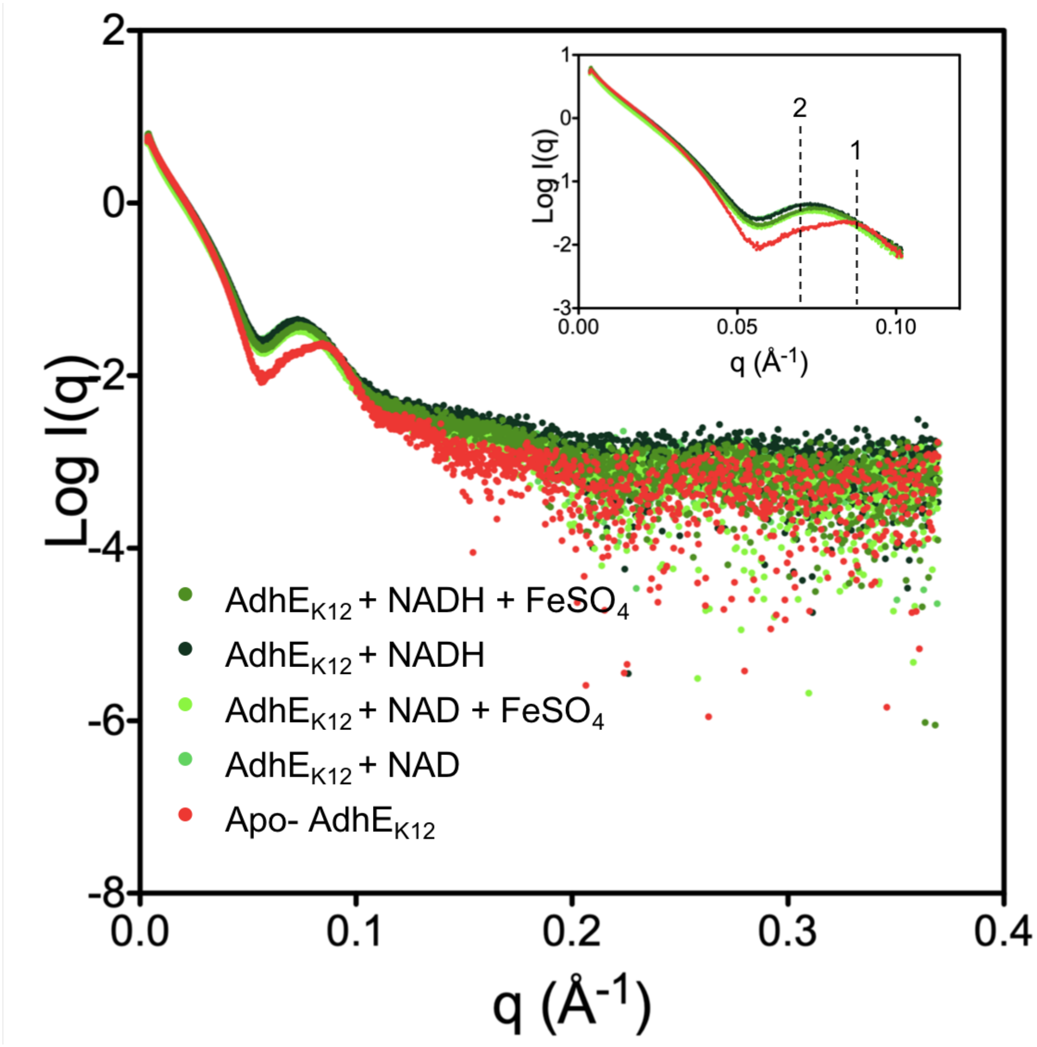

Supplementary Fig. 6. AdhE spirosomes change conformation in the presence of cofactors. SAXS data acquired in batch mode for AdhE fraction 2 in the presence (green) and absence (red) of cofactors reveal conformational changes evidenced by a shift in a conserved feature from q = 0.087 $Å^{-1}$ (indicated by 1 in the inset) for Apo-AdhE to q = 0.074 $Å^{-1}$ (2 in the inset) for AdhE + cofactors, which translates to 72.2 Å and 84.9 Å in real space.

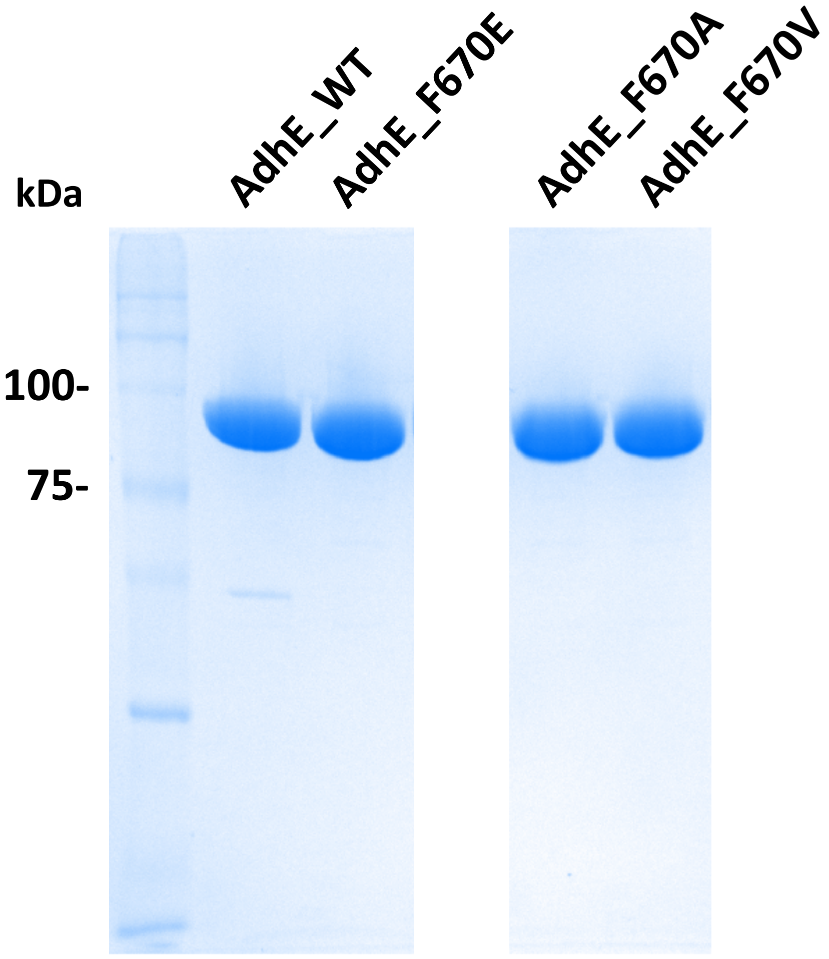

Supplementary Fig. 7. Purification of AdhE WT and mutants. SDS PAGE of WT and mutant AdhE. Molecular weight markers are in left-hand lane.

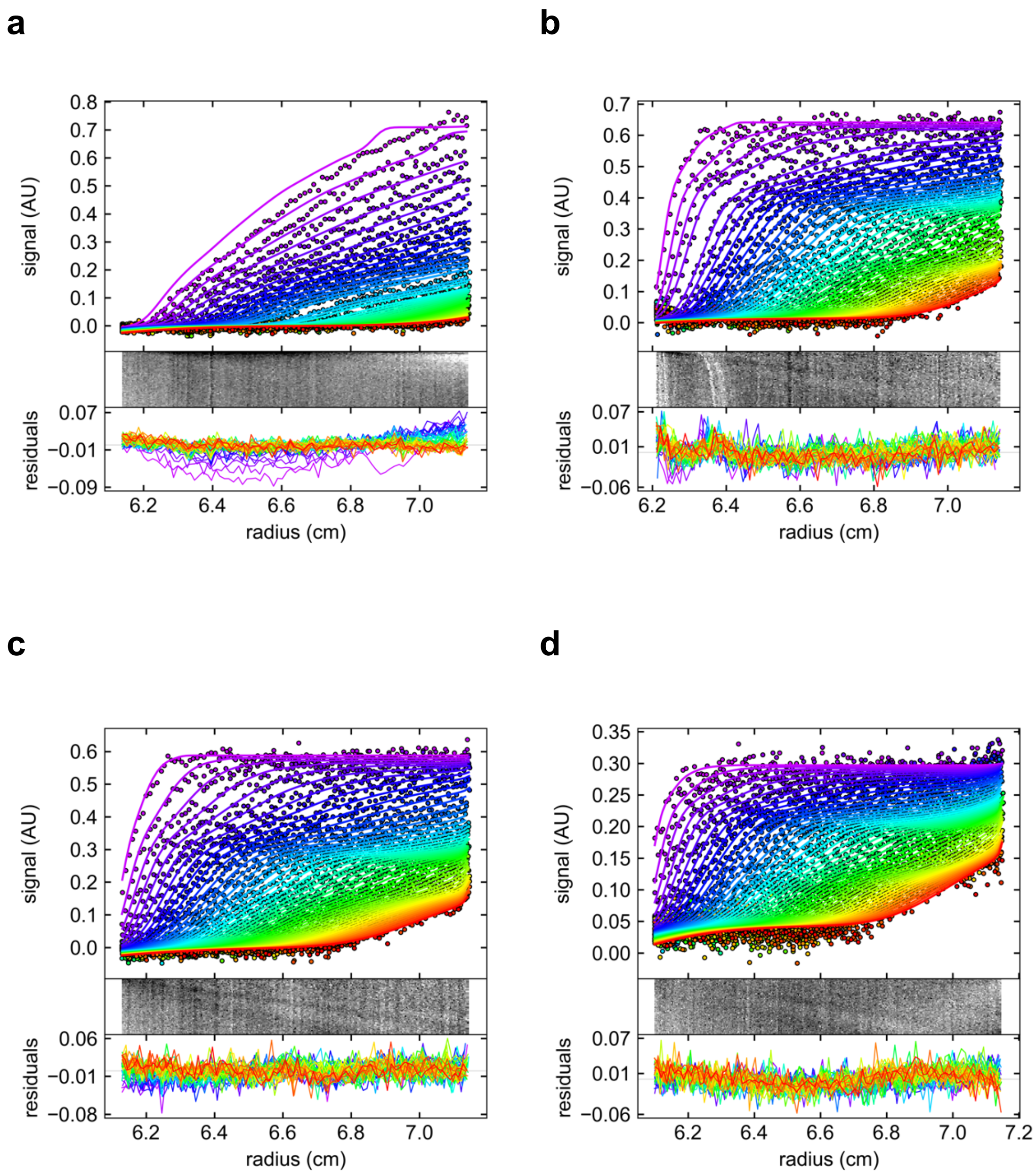

Supplementary Fig. 8. Primary AUC data and c(s) fits to the data that gave rise to Fig. 6e. Sedimentation velocity data acquired in absorbance mode for **a.** scans 5-55 for WT, and scans 1-50 for **b.** F670V, **c.** F670A, **d.** F670E AdhE (top panel), showing the fits to the data obtained using c(s) analysis together with the bitmap (middle panel, greyscale), and overlay (bottom panel, rainbow) of the residuals of these fits. The sums of the squares of residuals and root mean square deviations are: **a.** 2.69, 0.016; **b.** 3.17, 0.016; **c.** 3.00, 0.015, **d.** 3.27, 0.015.

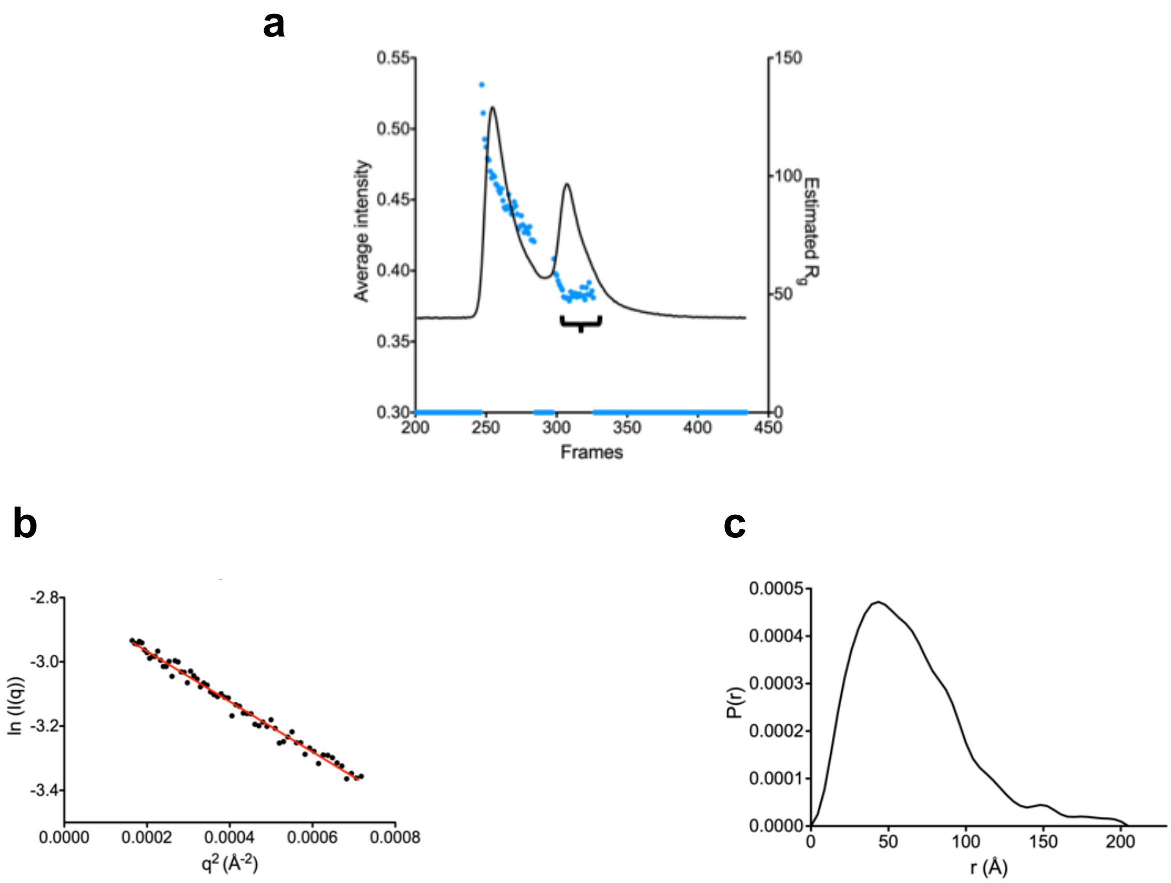

Supplementary Fig. 9. F670 mutants are polydisperse and include AdhE dimers and monomers. a. Integral ratio of signal (protein) to background (buffer) (black line) and estimated radius of gyration (Rg) (blue dots) plotted for each frame of data acquired; the bracket indicates the frames that were averaged and further analysed. **b.** Guinier and **c.** pairwise distance distribution analysis gave R_g = 48.4 Å and D_{max} = 205 Å, respectively.

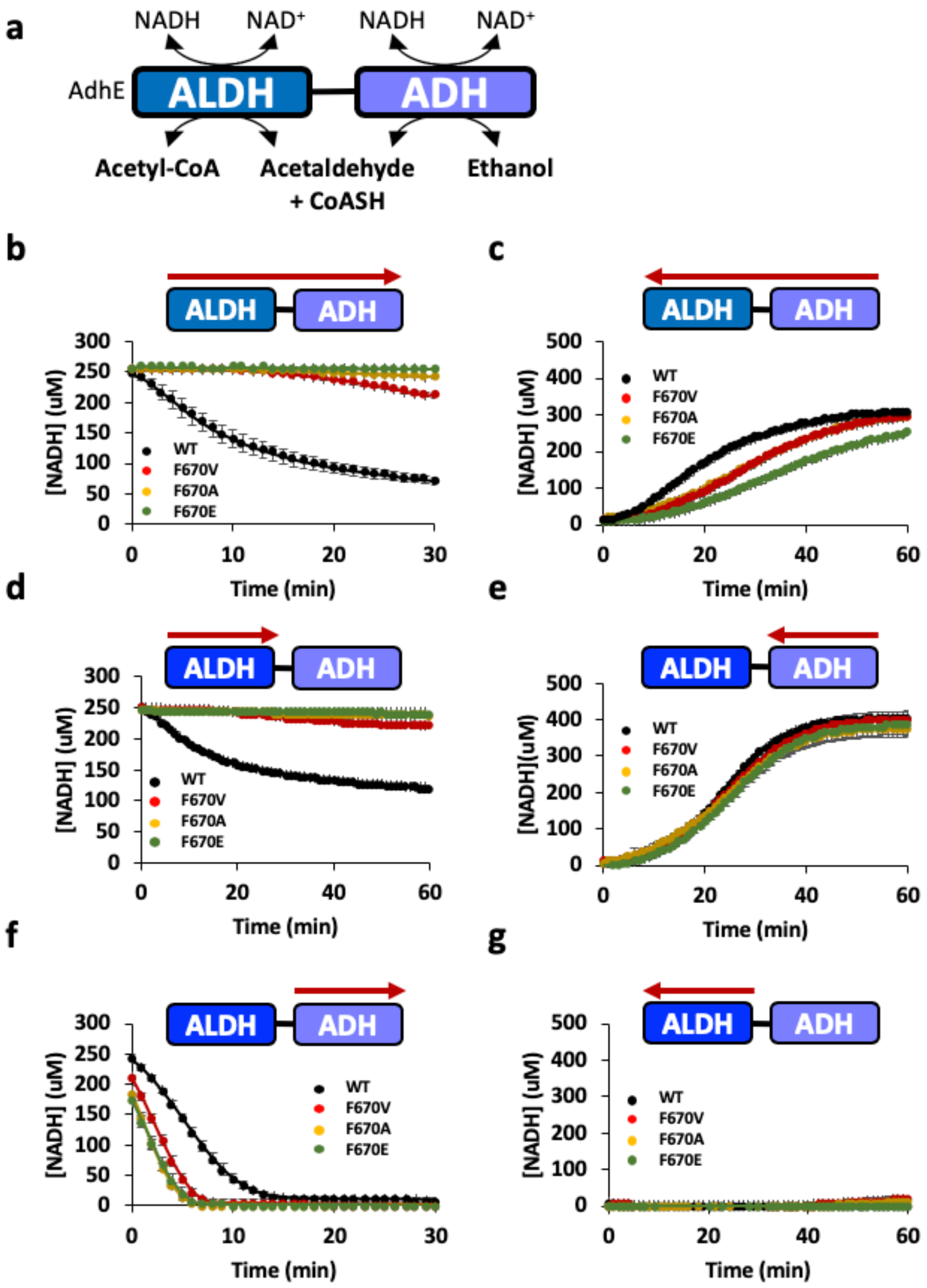

Supplementary Fig. 10. Enzymatic activity assay of the individual ALDH and ADH reactions. a. A scheme of the AdhE enzymatic reaction. **b.** Acetyl-CoA reductase (ALDH) and acetaldehyde reductase (ADH) activities of AdhE. **c.** Acetyl-CoA reductase (ALDH) activity. **d**. Acetaldehyde reductase (ADH) activity. **e.** Alcohol dehydrogenase (ADH^{-1}) and aldehyde ($ALDH^{-1}$) dehydrogenase activities. **f.** Alcohol dehydrogenase activity (ADH^{-1}). **g.** aldehyde dehydrogenase activity ($ALDH^{-1}$). The error bars show standard deviation (n=3).

Supplementary Table 1. Refinement statistics

	AdhE (fraction #2)	AdhE (fraction #1)
**Sample Preparation**		
Grid	Quantifoil R2/2 200 mesh	Quantifoil R2/2 300 mesh
Cryo-specimen freezing	Vitrobot IV	Vitrobot IV
**Data Collection**		
Electron Microscope	Titan Krios (300 keV)	Talos Artica (200 keV)
Detecting device	Falcon III (Electron counting mode)	Falcon III (Integration mode)
Total electron exposure/used (e/Å^2)	44.6/22.8	50/50
Defocus range (μm)	0.5-3.0	1.0-2.5
Pixel size (Å^2)	1.12	2.02
**Processing program**	cisTEM	RELION2.1
Obtained micrographs (no.)	1,255	652
Initial/Final particles used (no.)	251,604/160,830	43,151/39,443
Symmetry imposed	C1	Helical reconstruction
FSC threshold	0.143	0.143
**Resolution (Å)**	3.5	11.2
**Refinement program**	PHENIX	N.A.
**Model composition**		
Nonhydrogen atoms	46,106	
Protein residues	6,052	
B factors (Å^2)	162.49	
**R.m.s. Deviation**		
Bond Length (Å)	0.007	
Bond Angle (°)	1.28	
**Validation**	2.02	
MolProbity Score	1.61	
Clash Score	3.42	
Poor rotamers (%)	0.66	
**Ramachandran Plot**		
Favored (%)	92.2	
Allowed (%)	7.62	
Disallowed (%)	0.13	
**Mask CC**	0.80	

Supplementary Table 2: Partial specific volume, buffer density and viscosity calculated using SEDNTERP [2]

Calculated parameter	Temperature (°C)	4	20
Partial specific volume (ml/g)	Full-length AdhE expressed from *Yersina pestis*	0.735	0.741
	F670V, F670A & F670E and full-length AdhE expressed from *E. coli* K12		
Buffer density (g/ml)	50 mM HEPES, 500 mM NaCl, pH 7, 5% (v/v) glycerol	1.0387	0.01945
Buffer viscosity (Poise)		1.0369	0.01244

Supplementary Table 3: SAXS data collection and parameters

**Data collection**		
Instrument	SAXS B21 (Diamond light source)	
Wavelength (Å)	1	
q range ($Å^{-1}$)	0.0035 – 0.27	
Data	HPLC-SAXS; F670E dimer	BioSAXS – all AdhE scattering profiles
Exposure time (seconds)	3	1
Concentration range (mg ml^{-1})	9-10	3.8-5.2
Temperature (K)	277	298
**Structural parameters**		
I (0) (cm^{-1}) [from $P(r)$]	0.0608 ± 0.00197	-
R_g (Å) [from $P(r)$]	5.019 ± 0.2	-
I (0) (cm^{-1}) (from Guinier)	0.062 ± 0.00023	-
R_g (Å) (from Guinier)	5.012 ± 0.89	-
D_{max} (Å)	174	-
Porod volume estimate ($Å^3$)	106000	-
Dry volume calculated from sequence ($Å^3$)	116288	-
**Molecular mass determinant**		
Partial specific volume (cm^3g^{-1})	0.741	-
Molecular mass M_r [from I (0)]	199278	-
Calculated monomeric M_r from sequence	96127	-
**Software employed**		
Primary data reduction	SCATTER	
Data processing	SCATTER	-
Rigid-body modelling	SREFLEX	-
Computation of model intensities	FoXS	-
Three-dimensional graphics representation	PyMOL	-

Supplemental Table 4. Primers used in this study

Primer	Sequence (5’-3’)
adhE_forward	ATATGGATCCATGGCTGTTACTAATGTCGCTG
adhE_reverse	ATATCTCGAGTTAAGCGGATTTTTTCGCTTTTTTCTC
adhE_F670V_forward	GTA CTG GCA TCTGAGGTGTCTGATGGTCAGG
adhE_F670V_reverse	CCTGACCATCAGACACCTCAGATGCCAGTAC
adhE_F670A_forward	CTGGCATCTGAGGCGTCTGATGGTCAG
adhE_F670A_reverse	CTGACCATCAGACGCCTCAGATGCCAG
adhE_F670E_forward	CTGTACTGGCATCTGAGGAATCTGATGGTCAGGCTCTGCAG
adhE_F670E_reverse	CTGCAGAGCCTGACCATCAGATTCCTCAGATGCCAGTACAG

Supplementary References

1. Kucukelbir, A., Sigworth, F.J. & Tagare, H.D. Quantifying the local resolution of cryo-EM density maps. *Nature Meths* **11**, 63-65 (2014).
2. Hayes, D.B. et al. SEDNTERP. (2012).
